# Supplementary figures and images for: Time as a significant factor in the release of potassium from lithium heparin plasma and serum
Source: PLoS One. 2024 Dec 9;19(12):e0313572. doi: 10.1371/journal.pone.0313572 (PMC11627413; doi:10.1371/journal.pone.0313572)

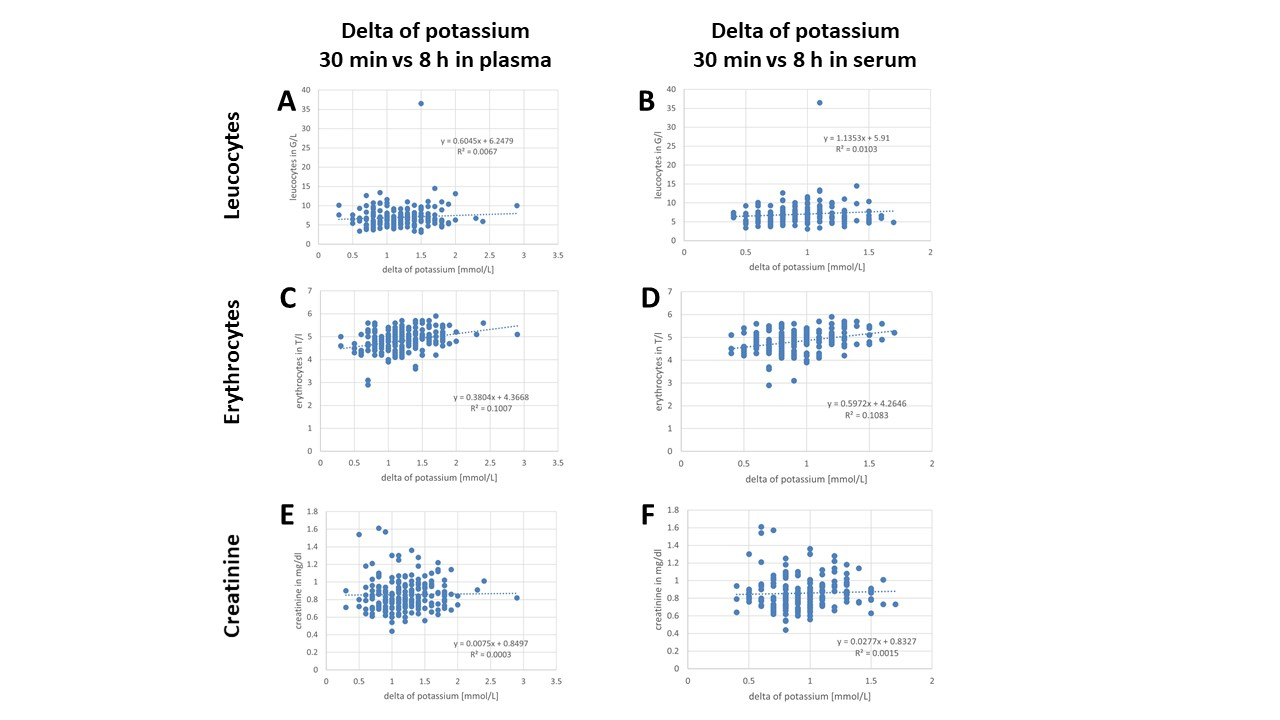

Supplement: S1 Fig — The difference between the potassium value after 8 h and 30 min is given as the delta for Li-Hep plasma (A, C, E) or serum (B, D, F). The potassium delta is set in relation to the influencing factors of the leukocyte (A, B), erythrocyte (C, D) and creatinine (E, F) measurement. R2 is given as an indication of the quality of the linear regression. There is no linear correlation between X and Y in these data. (TIFF) [file pone.0313572.s001.tiff]
